# Comparison of Clinical and Genetic Characteristics of Familial Mediterranean Fever Patients Among Adult Age Groups

**DOI:** 10.5152/tjg.2024.23662

**Published:** 2024-08-01

**Authors:** Sami Fidan, Sahile Seferli, Serdar Durak, Ceren Konca Seferoğlu, İlyas Ercan Okatan, Alper Han Çebi, Murat Erkut, Arif Mansur Coşar

**Affiliations:** 1 Department of Gastroenterology, Karadeniz Technical University Faculty of Medicine, Trabzon, Türkiye; 2 Department of Internal Medicine, Karadeniz Technical University Faculty of Medicine, Trabzon, Türkiye; 3 Department of Rheumatology, Karadeniz Technical University Faculty of Medicine, Trabzon, Türkiye; 4 Department of Medical Genetics, Karadeniz Technical University Faculty of Medicine, Trabzon, Türkiye

**Keywords:** Familial Mediterranean fever, adult onset FMF, MEFV mutation, FMF genotype

## Abstract

**Background/Aims:**

Familial mediterranean fever (FMF) is a genetic autoinflammatory disease typically diagnosed in childhood. In this study, we aimed to investigate the demographic, clinical, and genetic characteristics of patients aged 18 years and older who were diagnosed with FMF.

**Materials and Methods:**

Patients diagnosed with FMF between 2014 and 2022 at Karadeniz Technical University Faculty of Medicine Hospital were included in the study. Patients were divided into 2 groups based on the age of disease onset. Group I included patients with adult-onset (ages 18-40), while group II comprised patients with late onset (ages 40 and above). Subsequently, the 2 groups were compared.

**Results:**

A total of 150 patients with a mean age of 32 (18-79) were included in the study. There were 116 patients in group I and 34 (22.7%) in group II. The most common presenting complaint was abdominal pain (91.3%), and the most prevalent complication was amyloidosis (4.7%). No significant differences were observed between age groups regarding clinical findings and symptoms. The most frequent homozygous mutations were M694V (9.3%) and R202Q (1.8%), while the heterozygous mutations were M694V (37.3%) and R202Q (35.5%), respectively. The rate of M694V gene positivity in the adult-onset group was significantly higher compared to the late-onset group (52.9% and 25%, respectively, *P =* .020).

**Conclusion:**

**:** There does not appear to be a significant difference in clinical signs and symptoms between adult-onset and late-onset FMF patients. The higher rate of M694V gene positivity in the adult-onset group suggests that the M694V mutation may be responsible for the early expression of the disease.

**Main Points**

- Clinical and genetic information regarding familial Mediterranean fever (FMF) in adult patients is relatively limited.
- There are very few studies comparing adult- and late-onset FMF patients in terms of clinical and genetic findings.
- Our study suggests that there is no significant difference in clinical signs and symptoms between adult-onset FMF and late-onset FMF patients.
- The higher M694V gene positivity in the adult-onset group suggests that the M694V gene mutation may be responsible for the early onset of the disease.

## Introduction

Familial Mediterranean fever (FMF) is an autosomal recessive autoinflammatory disease. It is characterized by recurrent episodes of fever, abdominal pain, and serosal inflammation.^1,2^ Initially prevalent mainly in Sephardic Jews, Armenians, Turks, Arabs, Italians, Iranians, and Greeks, FMF has spread globally in recent years due to intense intercontinental migration.^3^ The estimated prevalence of the disease in Türkiye is around 1 in 1000.^4^ The disease is associated with mutations in the Mediterranean fever (MEFV) gene, located on the short arm of chromosome 16 (16p13.3). It encodes a 781-amino acid protein called pyrin, which has regulatory functions on the innate immune system.^5^ The precise structure, function, and role in triggering an inflammatory response are still not fully understood.^1^

There is no specific examination or laboratory test that can definitively diagnose FMF. Diagnosis is usually based on compatible clinical and laboratory findings, along with a positive family history. However, individuals with nonspecific symptoms, late-onset disease, or without a family history may face challenges in diagnosis. In such cases, mutation analysis can play a crucial role in providing an early diagnosis.^6^ Although attacks usually resolve spontaneously, without early diagnosis and appropriate treatment, patients can develop amyloidosis and kidney failure, which can become the leading cause of death in the later stages of the disease.^2^

While the first attack of FMF often occurs in childhood, the symptoms and findings of the disease may rarely manifest after the age of 40.^7^ As a result, studies of the disease mostly include patients diagnosed in childhood. In other words, there is relatively little clinical and genetic information available for FMF in adult patients. Previous studies have reported some clinical and genetic differences between adults (>18 years of age) and patients diagnosed in childhood; however, studies comparing adults with late-onset patients remain quite limited.^6-8^ In our study, we aimed to evaluate the sociodemographic characteristics, clinical findings, and MEFV mutation analysis results of patients aged 18 years and older diagnosed with FMF in our center. Additionally, we compared the adult and late-onset groups in terms of their clinical and genetic characteristics.

## Materials and Methods

### Study Design

This study included patients aged 18 years and older who were diagnosed with FMF at Karadeniz Technical University Faculty of Medicine Hospital between 2014 and 2022. The study was approved by the Ethics Committee of Karadeniz Technical University Faculty of Medicine (approval number: 24237859-673, date: September 15, 2021). No informed consent was needed because of the retrospective non-interventional study design. After obtaining the approval of the ethics committee and the permission of the chief physician of the hospital, the file numbers of patients with International Classification of Diseases (ICD) diagnosis codes for FMF were obtained from the hospital information system. Demographic characteristics at the time of initial diagnosis, date of diagnosis, coexisting diseases, clinical findings, laboratory, and MEFV gene analysis results, treatments received, follow-up period, and complications developed were retrospectively evaluated from both patient records and the hospital’s electronic database. The diagnosis of FMF was made according to the Tel Hashomer criteria.^9^ Patients were categorized into 2 groups based on age of symptom onset: adult-onset (group I, 18-40 years) and late-onset (group II, 40 years and older). The groups were compared in terms of clinical findings and genetic analysis results. Patients who did not fully meet the Livneh diagnostic criteria, were diagnosed before the age of 18 years, had a diagnosis of FMF but an unknown clinical course, or were not receiving any treatment, and were diagnosed with other periodic fever syndromes besides FMF during follow-up were excluded from the study.

### MEFV Mutation Analysis

For MEFV gene analysis, 2 mL of blood was collected from each patient. DNA extraction was performed using the MagNA Pure 24 system kit (Roche Diagnostic, UK). The analysis focused on the 20 most prevalent mutations observed in our country, employing the real-time polymerase chain reaction method on the Roche Light Cycler 480 instrument developed by TIB Molbiol (Roche Diagnostic, UK).

### Statistical Analysis

Statistical analyses were conducted using the Statistical Package for the Social Sciences Windows, version 22.0, software (IBM Corp., Armonk, NY, USA). Continuous variables were evaluated for normal distribution using histograms, Q-Q plots, and Shapiro–Wilk or Kolmogorov–Smirnov tests, depending on the sample size of the variable. Normally distributed continuous variables were presented as mean ± standard deviation throughout the study, and the independent samples *t*-test was used to compare the 2 groups. Other continuous variables were presented as median (minimum-maximum) values, and the non-parametric Mann–Whitney *U*-test was used to compare the groups. Categorical variables were presented as frequencies and percentages. Group comparisons for categorical variables utilized either Pearson’s chi-square test or Fisher’s exact test. Statistical significance was set at a *P*-value of .05 or less, with a 95% confidence interval.

## Results

A total of 150 patients participated in this study, including 103 females (68.7%) and 47 males (31.3%). Patients were divided into 2 groups based on age at disease onset: group I (18-40 years) and group II (40 years and older). Twenty-one patients (14%) had only the initial clinic visit without subsequent clinic visits. The median follow-up period for the 129 patients was 26.4 months (range: 0.8-134.7). Eight patients (5.3%) underwent an appendectomy. Treatment regimen included colchicine for 144 patients (96%), interleukin 1 antagonist for 2 patients, and anti-tumor necrosis factor alpha therapy for 1 patient. Comparisons between age groups revealed no significant differences in demographic characteristics, clinical findings, or complications. Detailed demographic and clinical characteristics of the enrolled patients are summarized in Table 1.

In our study, MEFV mutation analysis results were obtained for 109 (72.6%) of 150 patients diagnosed with FMF. Unfortunately, the MEFV mutation analysis results for the remaining 41 patients could not be retrieved from our hospital records. Among the analyzed patients, MEFV gene mutation was detected in 107 cases. Of these patients, 40 (37.3%) had heterozygous mutations in the M694V gene, 38 (35.5%) in the R202Q gene, 27 (25.2%) in the V726A gene, and 17 (15.8%) in the E148Q gene. Furthermore, 10 patients (9.3%) displayed homozygous mutations in the M694V gene, and 2 patients (1.8%) had homozygous mutations in the R202Q gene. No MEFV gene mutations were detected in 2 patients (1.9%).

In particular, the frequency of both homozygous and heterozygous M694V mutations was significantly higher in the adult group compared to the late-onset group. However, no significant differences were observed between the groups for other mutations. Detailed results of the MEFV gene mutation analysis for the patients are shown in Table 2.

When considering the initial symptoms and clinical findings associated with homozygous, heterozygous, and combined heterozygous MEFV gene positivity, our analysis revealed no significant differences between the groups with abdominal pain, fever, chest pain, or pericardial, skin, or renal involvement and amyloidosis (Table 3). Despite the presence of abdominal pain, amyloidosis, and renal and skin involvement in patients with the M694V mutation compared to those without, these differences were not statistically significant (Table 4).

## Discussion

In this study, we examined the clinical and genetic characteristics of adult patients diagnosed with FMF at our hospital, categorized based on the age of disease onset. A distinctive feature of FMF is that symptoms typically begin in childhood. Nearly 90% of patients experience their first attack before the age of 20.^10^ Therefore, studies in this context usually include patients diagnosed in childhood. In reality, however, clinical features of the disease are quite common in the adult population, which can present diagnostic challenges for clinicians. Recent studies have shown that late-onset patients are not as rare as previously thought.^7,11^ In this study, 77.3% of patients had their first attack between the ages of 18 and 40 years, while 22.7% were 40 years or older at the time of their first attack. Looking at the diagnosis rate over the age of 40, a similar result of 22.8% was reported in a study conducted in Japan involving 395 patients.^11^ Another Japanese study on 292 FMF patients found that 44 patients (15.1%) had their first attack after the age of 40.^7^ On the other hand, a study conducted in Iran on patients diagnosed with FMF after the age of 20 reported that 11.5% of patients were diagnosed after turning 40.^8^ In contrast to our findings, studies including a significant number of patients under the age of 18 have reported notably lower rates (ranging from 0.6% to 3.4%) of the initial diagnosis after the age of 40.^6,12-14^ The lower rates of patients diagnosed after the age of 40 in these studies may be due to differences in the study populations. While FMF is generally viewed as a disease affecting children and young adults, it should be considered in the differential diagnosis in the adult age group when appropriate clinical findings related to the disease are present.

In general, the reports suggest that patients diagnosed at a later age tend to have a milder clinical course compared to those diagnosed at an earlier age.^6,12,13,15^ In our study, however, we did not observe significant differences in symptoms, clinical findings, and complications between the group of patients aged 18-40 years and those aged 40 years and older. The most frequently observed symptoms in both groups were abdominal pain (90.5% and 94.1%), joint manifestations (64.7% and 55.8%), and fever (44.8% and 50%), respectively. In some previous studies conducted in Türkiye, certain findings such as arthralgia, arthritis, and erysipelas-like erythema were reported to be more common in patients with adult-onset FMF compared to those with earlier onset (<18-20 years).^11,12,15^ Similar findings have been reported in studies conducted in other countries.^6,7,16^ In a recent study conducted in Türkiye, similar to our findings, no significant difference was found between early-onset and late-onset FMF groups in terms of clinical manifestations, except for fever.^14^ In the adult population, our study suggests that there is no significant difference in clinical findings between the 18-40 and 40+ age groups.

The MEFV gene has 10 exons, and there are nearly 400 variants identified to date.^1^ Among the mutations that develop in this gene, V726A, M680I, E148Q, M694V, and M694I mutations constitute approximately 70%-80% of cases.^5^ However, none of the known mutations have been identified in approximately 10%-20% of clinically diagnosed FMF patients.^17^ In our study, MEFV gene mutations were detected in 107 patients. The most common heterozygous mutations were M694V (37.3%), R202Q (35.5%), V726A (25.2%), and E148Q (15.8%). Homozygous mutations were less frequent; M694V (9.3%) and R202Q (1.8%). A multicenter study including 1090 patients in Türkiye reported that the most common mutations as M694V (51.4%), M680I (14.4%), and V726A (8.6%).^12^ In a study conducted in Syria by Jarjour^18^ in 2010, among 153 patients, M694V was the most frequently observed gene mutation (36.5%), followed by V726A (15.2%), E148Q (14.5%), M680I (G/C) (13. 2%), and M694I (10.2%). In a large cohort of Armenian FMF patients consisting of 10,370 individuals, the most common mutations reported as M694V (41.34%), V726A (27.62%), and M680I (18.18%).^6^

In our study, we found no significant difference in terms of homozygous, heterozygous, and compound heterozygous (combined heterozygous, mixed alleles) MEFV gene positivity between the overall adult-onset group and the late-onset group. The M694V mutation is one of the most common of the approximately 400 genetic variations in the FMF gene. Homozygous M694V mutation is thought to be responsible for the development of amyloidosis and a more severe clinical course.^19,20^ Additionally, there are reports suggesting that M694V gene mutation may be a risk factor for early-onset disease.^7,15^ In our study, we observed a lower M694V gene mutation rate in the late-onset group compared to the adult-onset group (25% and 52.9%, respectively) (*P* = .020). Furthermore, although abdominal pain, amyloidosis, and kidney and skin involvement were more frequent in patients with the M694V gene mutation compared to those without, these differences were not statistically significant. Similar to our study, Kishida et al^7^ examined the M694V gene mutation rate in child, adult, and late-onset FMF patients and reported the M694V gene mutation rate significantly lower in the late-onset group (38.3%, 31.7%, and 18.2%, respectively) (*P* = .047). Pediatric patients carrying this mutation have been reported to exhibit an earlier age of onset and a more severe disease course compared to others.^21,22^ Similar findings have been reported in adult studies.^20^ Hence, close monitoring and early initiation of treatment are crucial to prevent potential complications, especially in patients found to be homozygous for the M694V gene.

Our study is one of the few to compare adult-onset and late-onset patient groups, providing important data on the clinical and genetic characteristics of the disease in the adult population. However, there are some limitations due to the retrospective nature of the study. A number of patients were excluded from the study due to incomplete clinical findings and symptom scores in their medical records. In addition, the absence of data on treatment outcomes in the medical records limited a detailed evaluation of treatment effectiveness. Moreover, MEFV gene mutation results were unavailable in the records of some patients, possibly obtained in other hospitals.

In summary, our study indicates that in the adult population, there is no significant difference in clinical findings and symptoms between patients diagnosed with FMF between the ages of 18-40 and those over 40. While the rate of M694V gene mutation is higher in the adult-onset group than in the late-onset group, clinically it does not result in a significant difference between the groups. Clinicians should not overlook FMF as a possible diagnosis based on the age of disease onset. Further studies are warranted to determine the clinical and genetic characteristics of FMF patients in the adult population.

## Figures and Tables

**Table 1.** Comparison of Demographic and Clinical Characteristics of Familial Mediterranean Fever Patients

Variable	All patients (n = 150)	**Adult-Onset Group** **Group I (18-39 years) ** **(n = 116, 77.3%)**	**Late-Onset Group** **Group II (≥40 years) ** **(n = 34, 22.7%)**	P*
Demographic characteristics				
Gender, male/female, n (%)		36 (31) / 80 (69)	11 (32.4) / 23 (67.6)	1
Age of diagnosis, median (min-max)	32 (18-79)	29 (18-39)	53.5 (40-79)	
FMF family history, n(%)	54 ( 51,9**)	39 (48.1)	15 (65.2)	.164
Follow-up period (month), median (min-max)	26.4 (0.8-134.7)	25,5 (0.8-134.7)	39.85 (2.9-103.4)	.189
Clinical findings, n (%)				
Abdominal pain	137 (91.3)	105 (90.5)	32 (94.1)	.733
Joint findings	94 (62.7)	75 (64.7)	19(55.9)	.421
Fever	69 (46)	52 (44.8)	17 (50)	.696
Chest pain	34 (22.7)	27 (23.3)	7 (20.6)	.820
Skin involvement	6 (4)	6 (5.2)	0	.338
Pericardial effusion	3 (2)	1 (0.9)	2 (5.9)	.129
**Complications, n** (%)				
Amyloidosis	7 (4.7)	4 (3.4)	3 (8.8)	.193
Vasculitis	7 (4.7)	6 (5.2)	1 (2.9)	1
Renal involvement	6 (4)	3 (2.6)	3 (8.8)	.130
Treatment n (%)				
Colchicine	144 (96)	113 (97.4)	31 (91.2)	.130
Other	3 (2)	1 (0.9)	2 (5.9)	.129

FMF, familial Mediterranean fever.

*Statistical analysis was performed between the groups. **Family history was questioned in 104 patients.

**Table 2.** Genetic Characteristics of Patients Undergoing Mutation Analysis for Familial Mediterranean Fever Based on Age Groups

Mutation	All Patients (n = 109) (n, %)	Adult-Onset Group Group I (18-39 years) (n, %)	Late-Onset Group Group II (≥40 years) (n, %)	P*
Homozygous	9	8 (9.4)	1 (4.2)	.680
Heterozygous	55	41 (48.2)	14 (58.3)	.489
Compound heterozygotes / complex allele	43	35 (41.2)	8 (33.3)	.637
No identifiable mutations	2	1 (1.2)	1 (4.2)	.393
The (+) ratio of each mutation (n, %)				
M694V (all)	51	45 (52.9)	6 (25)	.020
M680I (all)	20	15 (17.6)	5 (20.8)	.767
R202Q (all)	36	30 (35.3)	6 (25)	.463
M694V homozygous	11	10 (11.8)	1 (4.2)	.450
R202Q homozygous	7	6 (7.1)	1 (4.2)	1
M680I homozygous	2	1 (1.2)	1 (4.2)	.393
V726A homozygous	1	1 (1.2)	0	1
C605G homozygous	1	1 (1.2)	0	1
E167D homozygous	1	1 (1.2)	0	0
M694V heterozygous	39	35 (41.2)	4 (16.7)	.031
R202Q heterozygous	29	24 (28.2)	5 (20.8)	.604
E148Q heterozygous	19	14 (16.5)	5 (20.8)	.761
V726A heterozygous	27	17 (20)	10 (41.7)	.058
M680I heterozygous	18	14 (16.5)	4 (16.7)	1
P369S heterozygous	2	2 (2.4)	0	1
R408Q heterozygous	1	1 (1.2)	0	1
F479L heterozygous	1	1 (1.2)	0	1
G138G heterozygous	1	1 (1.2)	0	1
A165A heterozygous	1	1 (1.2)	0	1
A744S heterozygous	1	1 (1.2)	0	1
G304R heterozygous	1	1 (1.2)	0	1
M694I heterozygous	1	1 (1.2)	0	1
T267I heterozygous	1	1 (1.2)	0	1

*Statistical analysis was performed between the groups.

**Table 3.** Association of MEFV Gene Positivity with Presenting Symptoms and Clinical Findings

Variables, n (%)	Homozygous (+) (n = 9)	Heterozygous (+) (n = 55)	Combined Heterozygote, Mixed Alleles (+) (n = 43)	P*
Abdominal pain	8 (88.9)	48 (87.3)	40 (93)	.646
Appendectomy	0	0	3 (7)	.101
Fever	5 (55.6)	22 (40)	5 (11.6)	.086
Chest pain	3 (33.3)	16 (29.1)	0	.548
Pericardial involvement	0	3 (5.5)	0	.232
Joint findings	7 (77.8)	34 (61.8)	24 (55.8)	.458
Skin involvement	0	2 (3.6)	3 (7)	.581
Renal involvement	0	1 (1.8)	4 (9.3)	.172
Amyloidosis	0	1 (1.8)	4 (9.3)	.172
Vasculitis	0	4 (7.3)	3 (7)	.708

*Statistical analysis was performed to assess the presence of symptoms/clinical findings among patients with homozygous, heterozygous, compound heterozygous, and mixed allele gene positivity.

**Table 4.** M694V Gene Positivity and Clinical Characteristics

	M694V (−)	M694V (+)	P*
Gender, M/F, n (%)	15 (25.8)/43 (74.1)	20 (39.2)/31 (60.8)	.222
Abdominal pain	53 (91.3)	47 (92.2)	.544
Fever	26 (44.8)	22 (43.1)	1
Chest pain	14 (24.1)	10 (19.6)	.653
Pericardial involvement	3 (5.2)	0	.248
Joint findings	40 (69)	28 (54.9)	.243
Skin finding	2 (3.5)	4 (7.8)	.411
Renal involvement	1 (1.7)	3 (7.8)	.178
Amyloidosis	2 (3.5)	4 (7.8)	.411
Vasculitis	4 (6.9)	3 (5.9)	1
